# Machine learning-based differentiation of lung squamous cell carcinoma and adenocarcinoma using clinical-semantic and radiomic features

**DOI:** 10.3389/fonc.2025.1726193

**Published:** 2025-11-25

**Authors:** Yanju Li, Xiaomeng Yang, Yingjin Zhang, Jing Liang, Jiaxin Liu, Xinru Zheng, Jing Wang, Zhaoxiang Ye

**Affiliations:** 1 Tianjin Medical University Cancer Institute and Hospital, National Clinical Research Center for Cancer, Tianjin’s Clinical Research Center for Cancer, State Key Laboratory of Druggability Evaluation and Systematic Translational Medicine, Tianjin Key Laboratory of Digestive Cancer, Key Laboratory of Cancer Prevention and Therapy, Department of Radiology, Tianjin, China; 2 Tianjin University of Traditional Chinese Medicine, School of Public Health, Tianjin, China

**Keywords:** squamous cell carcinoma, adenocarcinoma, radiomics, machine learning, computed tomography

## Abstract

**Purpose:**

To evaluate and compare the predictive performance of machine learning methods using clinical-semantic, radiomic, and combined features in distinguishing squamous cell carcinoma (SCC) from adenocarcinoma (ADC) in non-small cell lung cancer (NSCLC).

**Methods:**

A total of 399 patients with pathologically confirmed NSCLC were retrospectively enrolled in 2017, and randomly divided into a training set (n=279) and a validation set (n=120). Clinical factors, semantic features, and radiomics features were collected and screened via the minimum redundancy maximum relevance (mRMR) method and least absolute shrinkage and selection operator (LASSO). We investigated 3 models constructed with 4 classifiers for histologic subtype prediction. The models were trained on the training cohort and their performance was evaluated on the independent validation cohort using accuracy, sensitivity, specificity, F1 score, precision and area under the receiver operating characteristic curve (AUC).

**Results:**

After feature selection, 10 representative features were finalized, comprising 4 clinical-semantic and 6 radiomic features. In the validation cohort, the support vector machine (SVM) classifier demonstrated promising predictive performance. When integrating clinical-semantic and radiomic features, the combined model (AUC = 0.871) showed potential in distinguishing NSCLC pathological subtypes, outperforming models based solely on clinical-semantic (AUC = 0.594) or radiomic features (AUC = 0.713). It achieved an accuracy of 0.892, a sensitivity of 0.758, a specificity of 0.943, a F1 score of 0.794, and a precision of 0.833. However, the AUC differences were not statistically significant, highlighting the need for further multi-center prospective validation.

**Conclusion:**

In this study, the SVM-based combined model, which integrated clinical-semantic and radiomic features, demonstrated promising performance among the four classifiers-based combined models in distinguishing between ADC and SCC. However, due to the study’s single-center, retrospective design and the lack of statistically significant differences in AUC for some models, the findings should be interpreted with caution. These results show potential but require future multi-center prospective validation before clinical application.

## 1 Introduction

Global cancer statistics released by GLOBOCAN in 2022 reveal that lung cancer remains the leading cause of both cancer incidence and mortality worldwide. In 2022, it was estimated that there were approximately 2.48 million new cases and over 1.8 million deaths attributed to this disease globally (1). Non-small cell lung cancer (NSCLC) accounts for approximately 85% of all lung cancer cases, with adenocarcinoma (ADC; 40–50%) and squamous cell carcinoma (SCC; 20–30%) constituting the two major histological subtypes (2). Studies have demonstrated substantial differences in the genetic and epigenetic profiles of ADC and SCC during tumorigenesis and progression (3, 4). Given the divergent therapeutic approaches for these subtypes (5, 6), rapid and accurate pathological classification is essential to ensure optimal clinical management.

In clinical practice, histological subtyping of NSCLC typically relies on tissue obtained through percutaneous biopsy. However, this invasive approach is contraindicated in patients with severe cardiopulmonary dysfunction, coagulation abnormalities, or poor tolerance to the procedure (7). Furthermore, due to the spatial and temporal heterogeneity of tumors, biopsy samples may fail to capture the full biological complexity of the lesion, potentially leading to sampling bias and inaccurate histological classification (3, 8). Therefore, the development of a safe, reliable, reproducible, and non-invasive method for pre-treatment histological subtype prediction holds significant clinical value for guiding personalized therapeutic strategies and prognostic assessment in patients with NSCLC.

Semantic features, defined as radiologists’ subjective interpretations based on computed tomography (CT) images, have been validated in multiple studies to correlate with lung cancer subtypes and pathological characteristics (9–11). Additionally, clinical variables such as age and gender contribute to the differentiation between ADC and SCC (12, 13). However, pathological subtyping based on semantic or clinical features suffers from inherent limitations, including clinician experience and sample heterogeneity, resulting in diagnostic inconsistencies that compromise the precision demanded by modern individualized therapeutic strategies.

With the exponential growth of medical imaging data, manual interpretation is confronted with challenges of inefficiency and high subjectivity. Radiomics, integrated with machine learning, enables the extraction of quantitative features from conventional images to construct predictive models, which can enhance the accuracy of diagnosis and prognosis and facilitate clinical decision-making (14, 15). Several studies have focused on the identification of histologic subtype of NSCLC based on radiomics. Zhu et al. (16) retrospectively analyzed 129 patients with NSCLC, and 485 radiomic features were derived from tumor regions labeled manually. Five features were chosen via logistic regression to establish a radiomics signature, with this signature achieving an area under the receiver operating characteristic curve (AUC) of 0.893 when validated in the test set. Yang et al. (17) retrospectively investigated the application of radiomics in NSCLC histologic subtype classification using three multicenter datasets. They extracted 788 radiomic features and developed predictive models through multiple feature selection strategies and classification algorithms. Results showed poor performance of models trained on single datasets, while models based on combined datasets had an average AUC of 0.78 in the test set. Wu et al. (18) enrolled 350 patients into two separate cohorts, with 440 radiomic features extracted per sample. Twenty-four feature selection methods and three classification methods were applied to identify SCC and ADC, with the Naive Bayes method achieving the maximum AUC of 0.72. Although these studies have achieved good results, the potential diagnostic value of semantic features and a comparison of different machine learning methods for distinguishing ADC from SCC has not been investigated.

Thus, this study aims to achieve two objectives: first, to evaluate and compare the predictive performance of clinical-semantic, radiomics, and combined models for distinguishing SCC from ADC in patients with NSCLC; second, to assess and compare the performance of four commonly used classifiers.

## 2 Materials and methods

### 2.1 Patients

This study retrospectively enrolled patients from Tianjin Medical University Cancer Institute and Hospital (TMUCIH) in 2017. Eligibility criteria were as follows: (1) pathologically confirmed ADC or SCC; (2) availability of contrast-enhanced CT images prior to treatment; and (3) a solitary tumor lesion >5 mm in diameter. Exclusion criteria were: (1) receipt of anti-tumor therapy before CT imaging; (2) history of other thoracic or systemic malignancies; (3) histologic subtypes other than ADC or SCC; and (4) incomplete clinical data or inadequate image quality. All patients’ images were collected using contrast-enhanced computed tomography (CE-CT).

### 2.2 Acquisition of clinical-semantic features

Two radiologists with over 5 years of experience in pulmonary nodule diagnosis independently assessed all pulmonary nodules on CT scans. Both radiologists were blinded to clinical information and pathological outcomes. Any discrepancies in interpretation were resolved through consensus discussion. The evaluated characteristics included: (1) age, (2) gender, (3) nodule location, (4) shape, (5) margin, (6) calcification, (7) cavitation, (8) air bronchograms, (9) pleural indentation, (10) vascular invasion, (11) lymph nodes, (12) long-axis diameter of the nodule, and (13) short-axis diameter of the nodule.

### 2.3 ROI segmentation and extraction of radiomics features

Regions of interest (ROIs) for pulmonary lesions were segmented semi-automatically using the Deepwise Multimodal Scientific Research Platform (version 2.5.2, https://keyan.deepwise.com) (Beijing Deepwise and League of PHD Technology Co., Ltd, Beijing, China) (19–22). Two radiologists assessed the segmentation outcomes and made manual adjustments to the delineations. Prior to radiomics feature extraction, the delineated CT data were resampled to a voxel size of 1 × 1 × 1 mm to standardize spatial resolution and enhance feature robustness.

Pyradiomics software (Version 3.0) was utilized for radiomics feature extraction, with the extracted features covering first-order features, shape features, grey level co-occurrence matrix (GLCM) features, grey level size zone matrix (GLSZM) features, grey level run length matrix (GLRLM) features, grey level dependence matrix (GLDM) features, and neighborhood grey tone difference matrix (NGTDM) features. In total, 1834 radiomic features were acquired following this procedure.

### 2.4 Features selection and prediction model establishment

The radiomic feature selection process was performed as follows. First, inter-scanner variability was assessed using the Mann-Whitney U test (23) with Benjamini-Hochberg (24) false discovery rate (FDR) correction, and features with an FDR-adjusted p < 0.05 were excluded. Subsequently, univariate analysis was conducted using the same test and correction method to identify features significantly associated with the outcome, and those with FDR-corrected p≥0.05 were further excluded. Based on the remaining features, the minimum redundancy maximum relevance (mRMR) algorithm was employed to reduce redundancy. Finally, least absolute shrinkage and selection operator (LASSO) regression with 5-fold cross-validation was applied to select the most predictive features.

For clinical-semantic feature selection, univariate analysis was first performed to exclude features irrelevant to the outcome variable (with a p-value≥0.05). Subsequently, inter-feature correlation analysis was conducted to eliminate highly correlated variables; meanwhile, the variance inflation factor (VIF) was used to further assess and address multicollinearity. Finally, the recursive feature elimination (RFE) algorithm was applied to screen out the clinical-semantic feature combination with the highest predictive value.

To distinguish between SCC and ADC, three types of predictive models were constructed using clinical-semantic, radiomics, and the combined features. Model development employed four commonly used machine learning classifiers, including LASSO, Random Forest (RF), support vector machine (SVM) and extreme gradient boosting (XGBoost). Prior to training the combined model, correlation analysis and multicollinearity testing were performed to ensure no significant correlation or collinearity between clinical-semantic and radiomics features. All models were trained and compared using consistent parameter settings.

### 2.5 Statistical analysis

Statistical analyses were performed using Python (Version 3.9.19). Independent samples t-test (25), Mann-Whitney U test, and chi-square test (26) were used to compare the characteristics of patients. Clinical feature selection was based on univariate and correlation analyses, with p-value < 0.05 considered statistically significant. The optimal machine learning model was selected by comparing performance metrics including AUC, accuracy, sensitivity, specificity, F1 score and precision. The DeLong test was used to assess pairwise differences in AUC between models and classifiers.

## 3 Results

### 3.1 Clinical characteristics of patients

A total of 399 patients with pathologically confirmed NSCLC were enrolled in this study, including 111 cases of SCC and 288 cases of ADC. All patients were randomly divided into a training cohort (n=279) and an internal validation cohort (n=120) at a ratio of 7:3. The age of the study population ranged from 30 to 79 years, with a mean age of 60 years. The clinical characteristics between SCC and ADC patients in training and validation sets are summarized and compared in **Table 1**. It shows that SCC patients were more likely to be elderly males with obvious symptoms and larger lesions, whereas ADC patients were more likely to be younger females without obvious symptoms and with smaller lesions (p < 0.05). Patient baseline characteristics in the training and validation sets are presented in **Supplementary Table 1**. All baseline characteristics were comparable between the training and validation cohorts (all P > 0.05), except for Air_Bronchograms (P = 0.022). However, this variable was excluded in subsequent feature selection steps and not involved in model construction and thus is unlikely to introduce bias into model development or validation.

**Table 1:** Comparison of clinical characteristics between SCC and ADC patients in training and validation sets.

Variables	Training set(n=279)			Validation set(n=120)		
	SCC (n=78)	ADC (n=201)	P value	SCC (n=33)	ADC (n=87)	P value
Age (years)	63.00 ± 6.50*	59.44 ± 8.53*	0.0060	60.18 ± 6.55*	58.06 ± 9.14*	P>0.05
Gender			P<0.001			0.0025
Male	67(85.90)	103(51.24)		29 (87.88)	49 (56.32)	
Female	11(14.10)	98(48.76)		4 (12.12)	38 (43.68)	
Long_Diameter (mm)	36.50 ± 20.00*	30.00 ± 18.00*	0.0002	37.61 ± 17.44*	31.00 ± 16.00*	P>0.05
Short_Diameter (mm)	27.00 ± 17.00*	23.00 ± 13.00*	0.0020	23.00 ± 18.00*	25.00 ± 15.00*	P>0.05
Location			P<0.001			P<0.001
Peripheral	43(55.13)	178(88.56)		14 (42.42)	79 (90.80)	
Central	35(44.87)	23(11.44)		19 (57.58)	8 (9.20)	
Shape			0.0043			0.0009
round/oval	26(33.33)	107(53.23)		5 (15.15)	44 (50.57)	
irregular	52(66.67)	94(46.77)		28 (84.85)	43 (49.43)	
Margin			0.0001			0.0004
Smooth	0(0.00)	2(1.00)		0(0.00)	0(0.00)	
Lobulated	53(67.95)	78(38.80)		27 (81.82)	38 (43.68)	
Spiculated	25(32.05)	121(60.20)		6 (18.18)	49 (56.32)	
Calcification			P>0.05			P>0.05
No	71(91.03)	182(90.55)		31 (93.94)	76 (87.36)	
Yes	7(8.97)	19(9.45)		2 (6.06)	11 (12.64)	
Cavitation			P>0.05			P>0.05
No	60(76.92)	158(78.61)		26 (78.79)	70 (80.46)	
Yes	18(23.08)	43(21.39)		7 (21.21)	17 (19.54)	
Air_Bronchograms			P<0.001			0.0074
No	30(38.46)	165(82.09)		12 (36.36)	57 (65.52)	
Yes	48(61.54)	36(17.91)		21 (63.64)	30 (34.48)	
Pleural_indentation			P<0.001			P<0.001
No	33(42.31)	17(8.46)		17 (51.52)	7 (8.05)	
Yes	45(57.69)	184(91.54)		16 (48.48)	80 (91.95)	
Vascular_invasion			P<0.001			0.0095
No	70(89.74)	122(60.70)		28 (84.85)	50 (57.47)	
Yes	8(10.26)	79(39.30)		5 (15.15)	37 (42.53)	
Lymph_nodes			P>0.05			P>0.05
No	56(71.79)	129(64.18)		28 (84.85)	58 (66.67)	
Yes	22(28.21)	72(35.82)		5 (15.15)	29 (33.33)	

ADC, adenocarcinoma; SCC, squamous cell carcinoma.

Data in parentheses are percentages unless otherwise noted.

*Values refer to mean ± standard deviation.

P values were the results of univariate analysis of each characteristic.

### 3.2 Features selection and prediction model establishment

**Figure 1:**
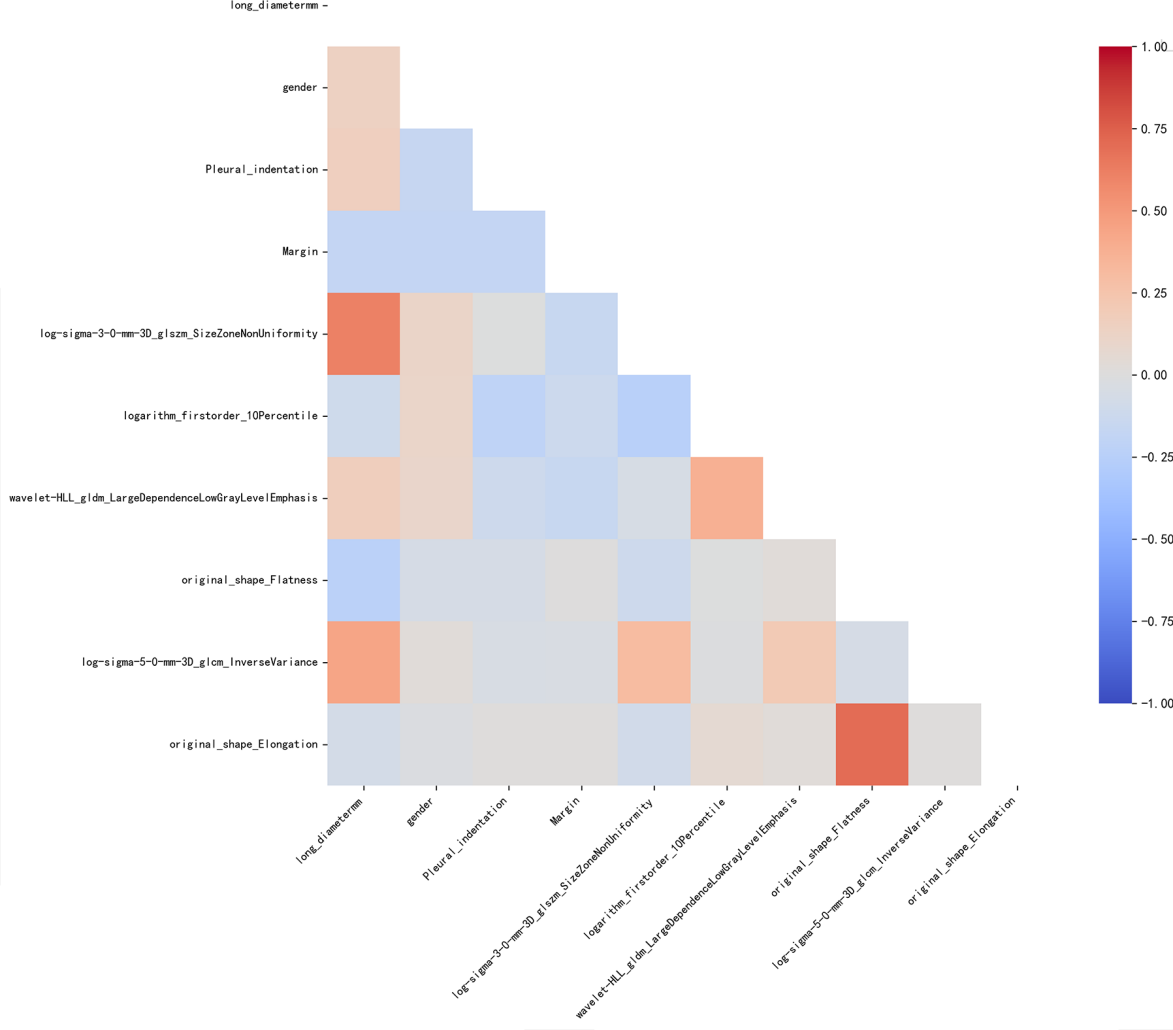
A correlation matrix among the final selected clinical-semantic and radiomics features.

After multi-step feature selection, 4 variables were finally identified in the clinical-semantic training cohort, and 6 representative features were selected from the radiomics training cohort. Correlation and multicollinearity analyses confirmed that no significant correlation or collinearity existed among these features. **Figure 1** presents the correlation matrix among the 10 candidate features. To avoid the interference of feature redundancy on model performance, a high correlation threshold was set as the absolute correlation coefficient |*r*|> 0.7; high-correlation feature pairs were screened, and the feature with weaker correlation to the target variable in each pair was eliminated. The analysis results showed that no high-correlation feature pairs meeting |*r*|> 0.7 were detected among the 10 features, so no features were removed. Based on these results, a combined model incorporating 10 features was developed in the training cohort. Detailed information on the selected 10 features is provided in **Table 2**.

**Table 2:** The final selected 10 clinical-semantic and radiomics features for clinical-semantic model, radiomics model and combined model.

Model		Features
combined model	clinical-semantic model	long_diameter_mm
		Margin
		Pleural_indentation
		gender
	radiomics model	log-sigma-3-0-mm-3D_glszm_SizeZoneNonUniformity
		logarithm_firstorder_10Percentile
		wavelet-HLL_gldm_LargeDependenceLowGrayLevelEmphasis
		original_shape_Flatness
		log-sigma-5-0-mm-3D_glcm_InverseVariance
		original_shape_Elongation

### 3.3 Prediction performance of classifiers

**Figure 2:**
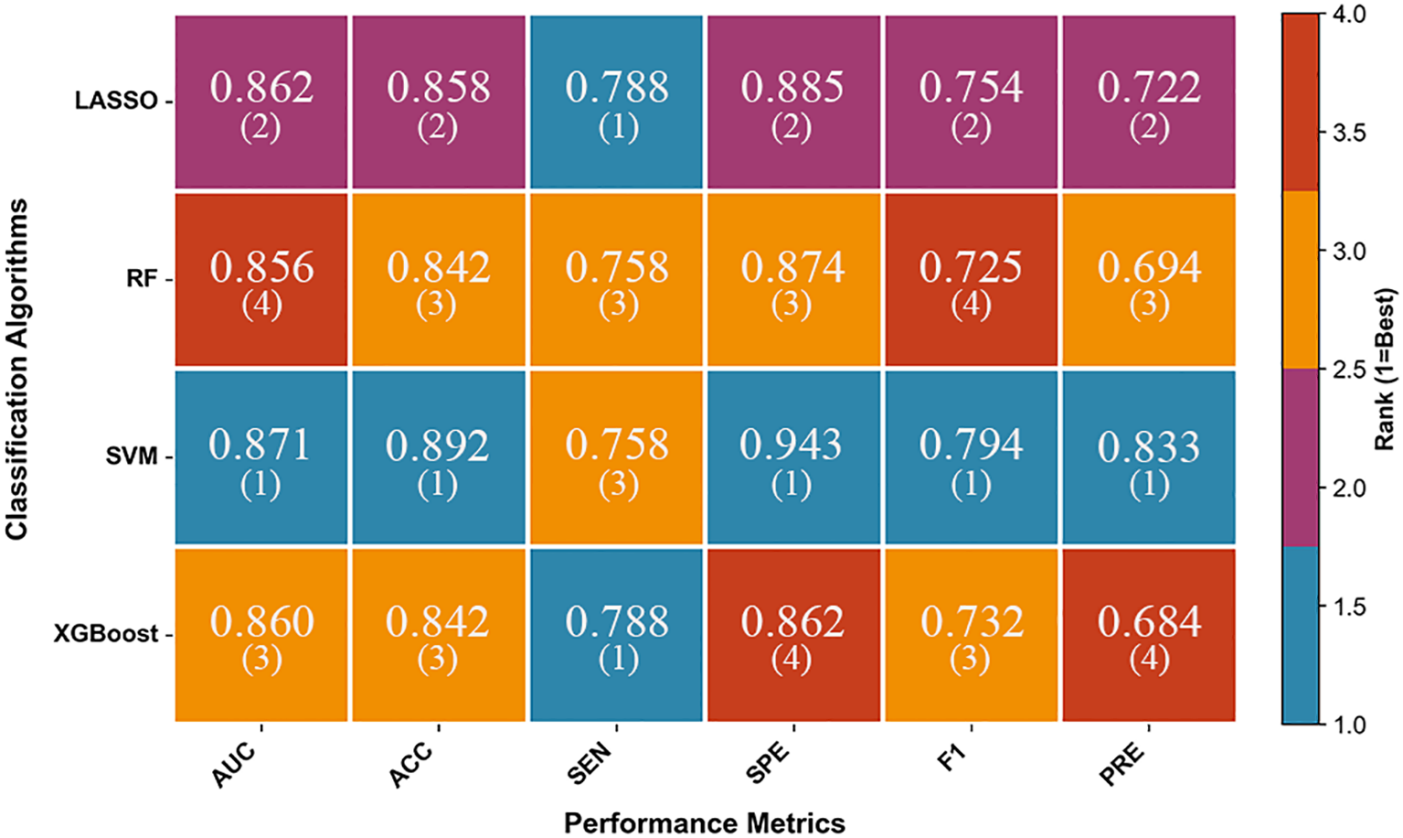
A ranked heatmap illustrating the performance of the four-classifier-based combined models in the validation cohort. LASSO, least absolute shrinkage and selection operator; RF, random forest; SVM, support vector machine; XGBoost, extreme gradient boosting; AUC, the area under the receiver operating characteristic curve; CI, confidence interval; ACC, accuracy; SEN, sensitivity; SPE, specificity; F1, F1 score; PRE, precision.

**Figure 2** presents a ranked heatmap illustrating the performance of the four-classifier-based combined models in the validation cohort. The x-axis represents six key performance metrics, while the y-axis lists the classifiers. Blue indicates the optimal performance, with the number “1” denoting the highest rank —smaller values correspond to better performance. The ranking heatmap visually demonstrates that the SVM-based combined model outperformed the LASSO, RF, and XGBoost combined models in five out of six key evaluation metrics. Specifically, the model demonstrated numerically optimal performance, achieving an AUC of 0.871, an accuracy of 0.892, a specificity of 0.943, an F1-score of 0.794, and a precision of 0.833. Although its sensitivity (0.758) was slightly lower than that of LASSO and XGBoost (both 0.788) and comparable to RF, the SVM-based combined model exhibited the best overall performance across all metrics among the four models. Pairwise comparisons of AUC among the four-classifier-based combined models via Delong test (with Holm-Bonferroni correction) showed that the vast majority of differences were not statistically significant (most corrected p > 0.05). The results of the Delong test are presented in **Supplementary Table 2**.

### 3.4 Prediction performance of prediction models

**Figure 3:**
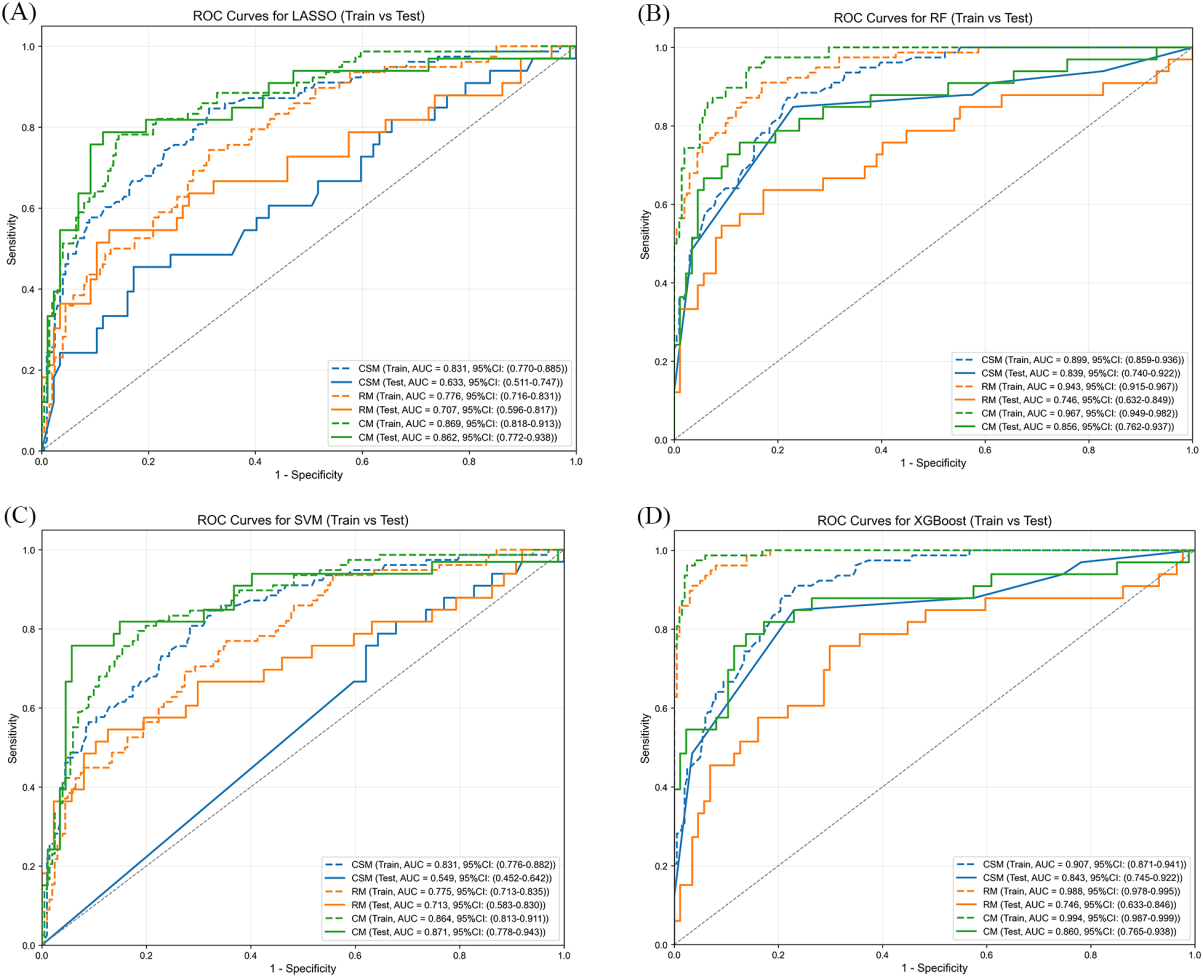
Receiver operating characteristic (ROC) curves of clinical-semantic, radiomics, and combined models constructed using four classifiers in training and validation cohorts. **(A)** ROC curves for the LASSO classifier (training vs. test). **(B)** ROC curves for the RF classifier (training vs. test). **(C)** ROC curves for the SVM classifier (training vs. test). **(D)** ROC curves for the XGBoost classifier (training vs. test). CSM, clinical-semantic model; RM, radiomics model; CM, combined model.

The Receiver operating characteristic (ROC) curves for the clinical-semantic, radiomics, and combined models, all constructed using four classifiers, in both the training and validation cohorts are presented in **Figure 3**. Identical hyperparameter settings were applied across the four classifiers to ensure that performance differences were primarily attributable to feature information rather than parameter tuning. Detailed hyperparameter configurations for each algorithm are provided in **Supplementary Table 3**. Overall, the combined model achieved the most favorable numerical performance in training and validation sets, outperforming the other two single-feature models in terms of AUC, accuracy, specificity, F1 score and precision. For instance, with the SVM classifier, (1) in the training cohort, the combined model yielded an AUC of 0.864 (95% confidence interval [CI], 0.813-0.911), an accuracy of 0.810, a specificity of 0.816, a F1 score of 0.701, and a precision of 0.626; (2) more importantly, in the validation cohort, which better reflects model generalizability, the combined model maintained a robust AUC of 0.871 (95% CI, 0.778-0.943), with an accuracy of 0.892, a specificity of 0.943, a F1 score of 0.794, and a precision of 0.833. All detailed results are presented in **Supplementary Table 4**.

To further assess the statistical significance of AUC differences among the three models using four classification algorithms, pairwise comparisons were performed using the DeLong test, with multiple testing corrections applied via the Holm-Bonferroni method. After correction, most P-values remained greater than 0.05, indicating that the majority of the observed differences in AUC were not statistically significant. Detailed results of the DeLong test are provided in **Supplementary Table 5**.

## 4 Discussion

In this study, we compared the ability of the four-classifier-based models to identify the pathological subtypes of NSCLC, and evaluated their predictive performance within clinical-semantic, radiomics, and combined modeling strategies. Results showed that the combined model constructed based on SVM algorithm exhibited the optimal comprehensive performance in distinguishing the histological subtypes of NSCLC.

A total of four representative clinical-semantic features were ultimately selected: long_diameter_mm (representing the maximum diameter of the lesion in the largest cross-sectional plane), margin, pleural_indentation, and gender. Our findings are consistent with previously reported trends (27–31): in addition to semantic characteristics such as margin irregularity, established clinical variables including male gender were more frequently associated with SCC. Furthermore, lesions in SCC patients were generally larger than those in ADC patients. Nevertheless, there were some differences in the present study: in our cohort, semantic features such as calcification, cavitation, and lymph nodes showed no significant differences between ADC and SCC, and did not contribute substantially to classifier performance.

In addition to clinical-semantic features, this study also analyzed radiomics features derived from CT images, and finally selected 6 representative features: log-sigma-3-0-mm-3D_glszm_SizeZoneNonUniformity, logarithm_firstorder_10Percentile, wavelet-HLL_gldm_LargeDependenceLowGrayLevelEmphasis, original_shape_Flatness, log-sigma-5-0-mm-3D_glcm_InverseVariance, and original_shape_Elongation. Previous studies by Bashir et al. (32)and Hyun et al. (33)have investigated the application of radiomics in NSCLC classification; however, the optimal feature sets identified in their studies included GLSZMSZLIE, coefficient of variation, NGTDM coarseness, and gray-level zone length nonuniformity, gray-level nonuniformity for zone. In contrast, the optimal radiomic subset identified in our study showed limited overlap with theirs. This discrepancy may arise from the large number of correlated radiomic features, where different high-level features may essentially reflect variations of the same underlying image characteristics.

In terms of predictive performance, this study demonstrated that the combined model integrating clinical-semantic features and CT radiomics features had a significantly higher AUC than models based solely on a single feature type. Zhang et al. (34)constructed three models using clinical features, PET/CT imaging features, and a combination of all features. Their results showed that the combined model (AUC = 0.870) outperformed both the clinical model (AUC = 0.848) and the radiomics model (AUC = 0.774). This finding is consistent with our results and further supports the value of multimodal feature integration in histological subtyping.

This study demonstrated that the SVM–based combined model achieved superior performance in distinguishing NSCLC histological subtypes. Previous studies have also explored the efficacy of different classification algorithms: Warkentin et al. (35) evaluated the ability of three machine learning models (XGBoost, RF, and LASSO) to predict the malignant risk of pulmonary nodules using cross-validation and grid search, with results showing that the LASSO model achieved the optimal predictive performance; Selvam et al. (36)applied 13 machine learning algorithms, including linear discriminant analysis, RF, and AdaBoost, for pulmonary nodule classification, and reported that a multilayer perceptron (MLP) classifier with ReLU activation achieved the highest accuracy (83%) in discriminating between SCC and ADC; Wu et al. (18) investigated radiomics-based prediction of ADC and SCC by comparing 24 feature selection methods and three classification algorithms, and showed that the Naive Bayes classifier performed best, with an AUC of 0.72. Building upon these prior findings, our study provides a systematic, head-to-head comparison of four commonly used classifiers under identical conditions—using the same dataset and feature selection pipeline—and demonstrates that the SVM-based combined model exhibits the most effective discriminative capability for this specific task. These results offer empirical evidence supporting SVM as a robust and reliable choice for radiomics-based histological subtype classification of NSCLC.

This study has several limitations. First, its single-center retrospective design may introduce selection bias; future multi-center prospective studies are needed to further evaluate and validate the model’s generalizability. Second, this study did not assess the inter- or intra-observer consistency of clinical-semantic features or ROI segmentation, despite their execution by experienced radiologists. The lack of quantitative reproducibility measuresibili as kappa statistics for semantic categorical features, Dice coefficients for segmentation concordance, and intraclass correlation coefficients for feature stabilityntsi introduce subjective variability, potentially affecting feature reliability and model robustness. Future work should employ standardized annotation protocols and quantify inter-reader agreement to reinforce the validity of both semantic and radiomic features. Third, although the combined model demonstrated the best numerical performance among all models, its superiority was not statistically significant in this cohort—likely due to limited sample size or effect size. Future studies with larger and more diverse datasets are needed to confirm its discriminative robustness. Finally, this study used machine learning algorithms for classification; in the future, attempts can be made to integrate deep learning techniques to optimize the performance of the classification model. In addition, this study conducted analysis based on CT radiomics features, whereas incorporating metabolic information from PET and other functional imaging modalities may enrich feature representation and enhance predictive accuracy.

In summary, this study developed a combined model based on the SVM algorithm that integrates clinical-semantic and radiomic features. This model demonstrates promising potential for noninvasively distinguishing pathological subtypes of NSCLC. With further validation in multi-center prospective studies, it has the potential to support clinical decision-making and facilitate personalized treatment planning.

## Data Availability

The original contributions presented in the study are included in the article/**Supplementary Material**. Further inquiries can be directed to the corresponding author.
