# Supplementary material for: Machine learning-based differentiation of lung squamous cell carcinoma and adenocarcinoma using clinical-semantic and radiomic features
Source: Front Oncol. 2025 Nov 25;15:1726193. doi: 10.3389/fonc.2025.1726193 (PMC12685679; doi:10.3389/fonc.2025.1726193)
Supplement: Supplementary file 1 [file Table1.docx]

Supplementary Material

# Supplementary Figures and Tables

## Supplementary Tables

**Supplementary Table 1.** Patient baseline characteristics in the training and validation sets .

| **Variables** | **Training set (n = 279)** | **Validation set (n = 120)** | **P value** |
| --- | --- | --- | --- |
| **Age (years)** | 61.0 ± 11.0* | 60.0 ± 11.0* | 0.130 |
| **Gender** |  |  | 0.512 |
| **Male** | 170(60.93) | 78(65.00) |  |
| **Female** | 109(39.07) | 42(35.00) |  |
| **Long_Diameter (mm)** | 32.0 ± 18.0* | 31.0 ± 18.0* | 0.719 |
| **Short_Diameter (mm)** | 24.0 ± 14.0* | 24.5 ± 16.0* | 0.682 |
| **Location** |  |  | 0.803 |
| **Peripheral** | 221(79.21) | 93(77.50) |  |
| **Central** | 58(20.79) | 27(22.50) |  |
| **Shape** |  |  | 0.251 |
| **round/oval** | 133(47.67) | 49(40.83) |  |
| **irregular** | 146(52.33) | 71(59.17) |  |
| **Margin** |  |  | 0.293 |
| **Smooth** | 2(0.72) | 0(0.00) |  |
| **Lobulated** | 131(46.95) | 65(54.17) |  |
| **Spiculated** | 146(52.33) | 55(45.83) |  |
| **Calcification** |  |  | 0.777 |
| **No** | 253(90.68) | 107(89.17) |  |
| **Yes** | 26(9.32) | 13(10.83) |  |
| **Cavitation** |  |  | 0.777 |
| **No** | 218(78.14) | 96(80.00) |  |
| **Yes** | 61(21.86) | 24(20.00) |  |
| **Air_Bronchograms** |  |  | 0.022 |
| **No** | 195(69.89) | 69(57.50) |  |
| **Yes** | 84(30.11) | 51(42.50) |  |
| **Pleural_indentation** |  |  | 0.727 |
| **No** | 50(17.92) | 24(20.00) |  |
| **Yes** | 229(82.08) | 96(80.00) |  |
| **Vascular_invasion** |  |  | 0.528 |
| **No** | 192(68.82) | 78 |  |
| **Yes** | 87(31.18) | 42 |  |
| **Lymph_nodes** |  |  | 0.350 |
| **No** | 185(66.31) | 86(71.67) |  |
| **Yes** | 94(33.69) | 34(28.33) |  |

Data in parentheses are percentages unless otherwise noted

*Values refer to mean ± standard deviation

**Supplementary Table 2**. Pairwise comparisons of AUC among the four-classifier-based combined models in the validation cohort (DeLong Test with Holm-Bonferroni correction).

| **Model 1** | **Model 2** | **AUC 1** | **AUC 2** | **AUC_Diff** | **Z_stat** | **P_value** | **Corrected_P** |
| --- | --- | --- | --- | --- | --- | --- | --- |
| LASSO | RF | 0.8617 | 0.8565 | 0.0052 | 0.5615 | 0.574432 | 1 |
| LASSO | SVM | 0.8617 | 0.8708 | -0.0091 | -2.914 | 0.003568 | 0.021408 |
| LASSO | XGBoost | 0.8617 | 0.8596 | 0.0021 | 0.199 | 0.842293 | 1 |
| RF | SVM | 0.8565 | 0.8708 | -0.0143 | -1.3007 | 0.193356 | 1 |
| RF | XGBoost | 0.8565 | 0.8596 | -0.0031 | -0.46 | 0.645505 | 1 |
| SVM | XGBoost | 0.8708 | 0.8596 | 0.0111 | 1.0075 | 0.313692 | 1 |

LASSO, least absolute shrinkage and selection operator; RF, random forest; SVM, support vector machine; XGBoost, extreme gradient boosting; AUC, the area under the receiver operating characteristic curve.

**Supplementary Table 3**. Hyperparameter configurations for the four [classification](https://www.bing.com/dict/search?q=classification&FORM=BDVSP6&cc=cn) algorithms.

| [classification](https://www.bing.com/dict/search?q=classification&FORM=BDVSP6&cc=cn) algorithm | Hyperparameter configuration |
| --- | --- |
| LASSO | penalty='l1', solver='liblinear', C=1.0, max_iter=5000,  class_weight='balanced', random_state=42 |
| Random Forest | n_estimators=100, max_depth=5,  min_samples_split=10, min_samples_leaf=5,  max_features='sqrt', class_weight='balanced',  random_state=42 |
| SVM | kernel='linear', C=1.0, probability=True,  class_weight='balanced', random_state=42 |
| XGBoost | n_estimators=100, max_depth=3, learning_rate=0.1,  subsample=0.8, colsample_bytree=0.8, reg_alpha=1, reg_lambda=3, gamma=0.1, scale_pos_weight=scale_pos_weight,  use_label_encoder=False,  eval_metric='logloss', random_state=42 |

**Supplementary Table 4**. Performance of three models constructed with four classification algorithms for differentiating pathological subtypes in the training and internal validation cohort.

| **Algorithm** | **Model** | **Cohort** | **AUC** | **95%CI** | **ACC** | **SEN** | **SPE** | **F1** | **PRE** |
| --- | --- | --- | --- | --- | --- | --- | --- | --- | --- |
| LASSO | clinical-  semantic | training | 0.831 | 0.770-  0.885 | 0.731 | 0.846 | 0.687 | 0.638 | 0.512 |
|  |  | validation | 0.633 | 0.511-  0.747 | 0.725 | 0.455 | 0.828 | 0.476 | 0.500 |
|  | radiomics | training | 0.776 | 0.716-  0.831 | 0.703 | 0.744 | 0.687 | 0.583 | 0.479 |
|  |  | validation | 0.707 | 0.596-  0.817 | 0.783 | 0.545 | 0.874 | 0.581 | 0.621 |
|  | combined | training | 0.869 | 0.818-  0.913 | 0.835 | 0.782 | 0.856 | 0.726 | 0.678 |
|  |  | validation | 0.862 | 0.772-  0.938 | 0.858 | 0.788 | 0.885 | 0.754 | 0.722 |
| RF | clinical-  semantic | training | 0.899 | 0.859-  0.936 | 0.806 | 0.872 | 0.781 | 0.716 | 0.607 |
|  |  | validation | 0.839 | 0.740-  0.922 | 0.792 | 0.848 | 0.770 | 0.691 | 0.583 |
|  | radiomics | training | 0.943 | 0.915-  0.967 | 0.853 | 0.910 | 0.831 | 0.776 | 0.676 |
|  |  | validation | 0.746 | 0.632-  0.849 | 0.775 | 0.636 | 0.828 | 0.609 | 0.583 |
|  | combined | training | 0.967 | 0.949-  0.982 | 0.882 | 0.949 | 0.856 | 0.818 | 0.718 |
|  |  | validation | 0.856 | 0.762-  0.937 | 0.842 | 0.758 | 0.874 | 0.725 | 0.694 |
| SVM | clinical-  semantic | training | 0.831 | 0.776-  0.882 | 0.742 | 0.808 | 0.716 | 0.636 | 0.525 |
|  |  | validation | 0.549 | 0.452-  0.642 | 0.475 | 0.788 | 0.356 | 0.452 | 0.317 |
|  | radiomics | training | 0.775 | 0.713-  0.835 | 0.717 | 0.692 | 0.726 | 0.578 | 0.495 |
|  |  | validation | 0.713 | 0.583-  0.830 | 0.783 | 0.545 | 0.874 | 0.581 | 0.621 |
|  | combined | training | 0.864 | 0.813-  0.911 | 0.810 | 0.795 | 0.816 | 0.701 | 0.626 |
|  |  | validation | 0.871 | 0.778-  0.943 | 0.892 | 0.758 | 0.943 | 0.794 | 0.833 |
| XGBoost | clinical-  semantic | training | 0.907 | 0.871-  0.941 | 0.806 | 0.910 | 0.766 | 0.724 | 0.602 |
|  |  | validation | 0.843 | 0.745-  0.922 | 0.792 | 0.848 | 0.770 | 0.691 | 0.583 |
|  | radiomics | training | 0.988 | 0.978-  0.995 | 0.932 | 0.962 | 0.920 | 0.886 | 0.824 |
|  |  | validation | 0.746 | 0.633-  0.846 | 0.717 | 0.758 | 0.701 | 0.595 | 0.490 |
|  | combined | training | 0.994 | 0.987-  0.999 | 0.971 | 0.962 | 0.975 | 0.949 | 0.938 |
|  |  | validation | 0.860 | 0.765-  0.938 | 0.842 | 0.788 | 0.862 | 0.732 | 0.684 |

LASSO, least absolute shrinkage and selection operator; RF, random forest; SVM, support vector machine; XGBoost, extreme gradient boosting; AUC, the area under the receiver operating characteristic curve; CI, confidence interval; ACC, accuracy; SEN, sensitivity; SPE, specificity; F1, F1 score; PRE, precision.

**Supplementary Table 5**. Pairwise Comparisons of AUC among the four-classifier-based models in the validation cohort (DeLong Test with Holm-Bonferroni correction).

|  | **Model1** | **Model2** | **AUC1** | **AUC2** | **AUC_Diff** | **Z_stat** | **P_value** | **Corrected_P** |
| --- | --- | --- | --- | --- | --- | --- | --- | --- |
| LASSO | CM | CSM | 0.8617 | 0.6327 | 0.2290 | 2.4874 | 0.012869 | 0.038607 |
|  | CM | RM | 0.8617 | 0.7071 | 0.1546 | 1.8252 | 0.067978 | 0.203934 |
|  | CSM | RM | 0.6327 | 0.7071 | -0.0744 | -0.7488 | 0.453970 | 1 |
| RF | CM | CSM | 0.8565 | 0.8386 | 0.0179 | 0.2365 | 0.813076 | 1 |
|  | CM | RM | 0.8565 | 0.7464 | 0.1101 | 1.3389 | 0.180588 | 0.541765 |
|  | CSM | RM | 0.8386 | 0.7464 | 0.0921 | 1.0432 | 0.296878 | 0.890634 |
| SVM | CM | CSM | 0.8708 | 0.5495 | 0.3213 | 3.1780 | 0.001483 | 0.004449 |
|  | CM | RM | 0.8708 | 0.7133 | 0.1574 | 1.8923 | 0.058454 | 0.175362 |
|  | CSM | RM | 0.5495 | 0.7133 | -0.1639 | -1.5203 | 0.128427 | 0.385280 |
| XGBoost | CM | CSM | 0.8596 | 0.8434 | 0.0162 | 0.2172 | 0.828089 | 1 |
|  | CM | RM | 0.8596 | 0.7457 | 0.1139 | 1.3697 | 0.170783 | 0.512350 |
|  | CSM | RM | 0.8434 | 0.7457 | 0.0977 | 1.0860 | 0.277477 | 0.832432 |

LASSO, least absolute shrinkage and selection operator; RF, random forest; SVM, support vector machine; XGBoost, extreme gradient boosting; CSM, clinical-semantic model; RM, radiomics model; CM, combined model; AUC, the area under the receiver operating characteristic curve.
